# Strengthening the EU Health Technology Assessment Regulation: Integrating National Immunization Technical Advisory Groups for Comprehensive Vaccine Assessments

**DOI:** 10.3390/jmahp13020016

**Published:** 2025-04-18

**Authors:** Jasmijn Beekman, Adrianne de Roo, Sharon Wolters, Ramesh Marapin, Gabriel Gurgel do Amaral, Evgeni Dvortsin, Sibilia Quilici, Chiara de Waure, Elena Petelos, Maarten Postma, Anna Viceré

**Affiliations:** 1 Asc Academics, 9725 AK Groningen, The Netherlands; jasmijn.beekman@ascacademics.com (J.B.); sharon.wolters@rug.nl (S.W.); gabriel.gurgel@ascacademics.com (G.G.d.A.); evgeni@ascacademics.com (E.D.); 2 Valneva Austria GmbH, 1030 Vienna, Austria; adrianne.deroo@valneva.com; 3 Department of Health Sciences, University Medical Center Groningen, University of Groningen, 9713 GZ Groningen, The Netherlands; m.j.postma@rug.nl; 4 Department of Neurology, University Medical Center Groningen, University of Groningen, 9713 GZ Groningen, The Netherlands; r.s.marapin@umcg.nl; 5 Vaccines Europe, 1000 Brussels, Belgium; sibilia.quilici@vaccineseurope.eu; 6 Department of Medicine and Surgery, University of Perugia, 06123 Perugia, Italy; chiara.dewaure@unipg.it; 7 Clinic Social and Family Medicine, School of Medicine, University of Crete, 70013 Heraklion, Greece; elena.petelos@med.uoc.gr; 8 Health Services Research, Care and Public Health Research Institute, Faculty of Health, Medicine and Life Sciences, Maastricht University, 6211 LK Maastricht, The Netherlands; 9 Department of Economics, Econometrics & Finance, Faculty of Economics and Business, University of Groningen, 9700 AB Groningen, The Netherlands; 10 Centre of Excellence in Higher Education for Pharmaceutical Care Innovation, Universitas Padjadjaran Bandung, Jatinagor 40132, Indonesia; 11 Division of Pharmacology & Therapy, Faculty of Medicine, Universitas Airlangga, Surabaya 60115, Indonesia

**Keywords:** health technology assessment, NITAGs, EU HTAR, joint clinical assessment, vaccines, access, public health

## Abstract

Background: Given their crucial role in vaccine assessment, National Immunization Technical Advisory Groups (NITAGs) should be considered in the Regulation on Health Technology Assessment (EU HTAR) to maximize the benefits of the EU HTAR for vaccines. This review and perspective piece identifies the gaps arising from NITAGs potential lack of involvement and proposes strategies for involving them. Methods: A targeted literature and guideline review was conducted to evaluate NITAGs’ current and future role in relation to the EU HTAR. The impact of the EU HTAR on diverse national HTA frameworks was explored in a three-country case study. Recommendations were developed to leverage strengths and address weaknesses to ensure consistent and cohesive vaccine assessments. Results: The case study revealed potential overlaps between NITAGs and the EU HTAR, particularly regarding horizon scanning and joint scientific consultations. The involvement of NITAGs in national assessments varies, influencing how well joint clinical assessment reports will ultimately align with and be applicable to individual Member States. Conclusions: Stronger consideration of vaccines within the EU HTAR and NITAG involvement can streamline assessments, reduce duplication, and improve alignment between European and national processes. Strategic actions, including capacity building and collaborations between NITAGs, are key in facilitating this process.

## 1. Introduction

It is integral for a well-functioning healthcare system to have an effective, evidence-based, cost-effective, and safe immunization program as the principal public health measure for protecting populations against infectious diseases [1,2,3]. For Member States (MSs) of the European Union (EU), population-wide access to vaccines is generally secured via publicly funded National Immunization Programs (NIPs), and vaccine inclusion in a NIP is determined at the MS level, with the support and guidance of several expert bodies and stakeholders [3]. Vaccines are regulated as medicinal products in the EU, but market access pathways and decision-making differ from those for therapeutic products because of their preventive character. Additionally, this distinction stems from the fact that vaccines are not just individual treatments but essential pillars of public health used to protect populations against infectious diseases. Thus, they require a distinct framework and expertise to encompass and reflect many of the clinical, economic, and societal benefits of vaccines and population-wide immunization [3,4,5,6].

To better inform and develop evidence-based immunization policies, the Global Vaccine Action Plan 2011–2020 of the World Health Organization (WHO) called for all countries to establish or to have access to a National Immunization Technical Advisory Group (NITAG) by 2020 [2]. Present in all EU countries, a NITAG is an independent, multidisciplinary body consisting of national experts that provides evidence-based vaccine recommendations to policymakers and immunization program managers. The core tasks of a NITAG are to offer independent technical assistance and to provide advice and support for decision-makers. They evaluate evidence using tools such as health technology assessment (HTA) to advise on vaccine inclusion in the NIP, amongst others [3,7]. However, exact NITAG roles, activities, and outcomes vary across the EU countries, including when it comes to their use of HTA frameworks and methodologies. Of the 27 EU MSs, only 7 NITAGs follow the guidance of a published decision-making framework [3,8]. This results in variation and discrepancies in vaccine assessment and subsequent inclusion in the NIPs across the EU countries, and ultimately can impact the population’s access to vaccines [9]. In 2018, the European Centre for Disease Prevention and Control (ECDC) launched the EU/European Economic Area (EEA) NITAGs collaboration network—a comprehensive EU/EEA-wide system for sharing existing and scientific research on vaccines and facilitating the joint generation and dissemination of current scientific evidence [10].

In 14 of the 27 EU MS, NITAGs coexist with HTA bodies (HTAbs), which assess vaccines in parallel or in conjunction with NITAGs to provide recommendations to inform vaccine pricing and reimbursement. However, significant differences exist in the number and type of vaccine-specific criteria currently being used to evaluate vaccines by NITAGs and HTAb [11]. HTAbs often evaluate vaccines using non-vaccine-specific methodologies and processes, contributing to variability in the content of assessment reports across EU MSs [3]. Where NITAGs coexist with HTAbs, NITAG recommendation timelines tend to lengthen, while the time from recommendation to funding and from marketing authorization to population access tends to shorten [3,4,12]. In most cases, the final decision by decision-makers (often the Ministry of Health) aligns with the NITAG advice [7]. This demonstrates the important role that NITAGs play in the assessment of vaccines at the national level.

To improve the availability of innovative health technologies and strengthen the quality of HTAs across the EU, the Regulation (EU) 2021/2282 on Health Technology Assessment (EU HTAR) was adopted on 11 January 2022 and came into force on 12 January 2025 [13,14]. In support of the EU HTAR, six implementing acts will be published to provide more detailed rules for the application of the EU HTAR legislation [14]. The new EU HTAR creates an EU framework for the clinical assessment of health technologies encompassing medical products, including vaccines, by fostering collaboration and coordination between EU MSs. Its goals are to support national authorities in making more timely and informed decisions on the pricing and reimbursement of health technologies, streamline the procedures for health technology developers, and ensure wider patient access to health technologies [15]. The EU HTAR focuses on the clinical aspects of HTA, i.e., the relative clinical effectiveness and relative clinical safety of a new health technology as compared with existing technologies. Under the EU HTAR, the HTA coordination group and its subgroups, composed of national HTA bodies [13], will collaborate in four activities: horizon scanning, joint scientific consultation (JSC), joint clinical assessment (JCA), and voluntary cooperation on HTA among MSs. Horizon scanning will help MSs to identify, at an early stage, promising health technologies, to help health systems prepare for them. The JSC offers non-binding scientific advice for health technology developers, with the main objective of providing unified recommendations for HTAbs regarding drugs or medical device development. The JCA begins with PICO (population; intervention; comparator; outcomes) scoping and results in a JCA dossier produced by the manufacturer. JCAs will run parallel to the European Medicines Agency (EMA) assessment. Voluntary cooperation on HTA entails that the European Commission (EC) will support the cooperation among MSs on relevant processes that may extend beyond the scope of the EU HTAR, including the non-clinical assessment of health technologies.

The EU HTAR’s application starts gradually with oncology medicinal products and Advanced Therapy Medicinal Products (ATMPs) in 2025, with the plans to assess selected high-risk medical products from 2026 onwards, adding orphan medicinal products in 2028, and including all other medicinal products, encompassing vaccines, from 2030 onwards. With significant efforts ongoing to make the EU HTAR operational, the focus has been on the health technologies subject to the JCAs as of 12 January 2025. As of March 2025, three implementing acts have been adopted, two draft implementing acts have been published, and one implementing act remains under development. These implementing acts do not yet discuss any vaccine-specific methods, despite the HTAR directly referring to them in article 4 [13].

Vaccines may be included in JSCs, horizon scanning, and voluntary collaboration activities from 2025 onwards [13], but their specifics have unfortunately not yet received due consideration. As the legislation has already been implemented, there are currently no opportunities for changes. However, while the planned 2028 evaluation is meant for the EU HTAR to date, it does present an opportunity to enhance vaccine-specific assessments within the fully applicable framework. However, proactive efforts are needed now, as vaccine trial design and clinical development, not to mention the generation of real-world data and evidence synthesis, can take many years and are even longer than for other therapeutic medicines. Experts from regulatory agencies, the industry and public health organizations emphasize the importance of open dialogue, particularly regarding the involvement of NITAGs, to ensure a well-informed and collaborative approach on the applicability of the EU HTAR to vaccines. Critically, governance aspects at national/MS and supranational/cross-border levels ought to be carefully planned, considered, and implemented. Organizational and institutional changes are typically slow to implement, requiring thorough preparation. Additionally, potential legislative and procedural issues must be addressed at the MS level, such as the mandates of NITAGs or national HTAbs. Taking timely action will enable stakeholders to align evidence generation with NITAG requirements, facilitating efficient processes and ensuring vaccine access is guided by scientific rigor rather than administrative barriers.

This perspective piece and literature review explored how NITAGs’ knowledge and expertise can support the implementation of the EU HTAR and developed recommendations for their involvement under the Regulation. In a paper by Largeron et al. (2024), the authors put forth 13 recommendations under three guiding principles to support the effective implementation of EU HTAR for vaccines [3]. The second of these principles, i.e., “develop inclusive, timely, and transparent vaccine assessment processes to support stronger evidence generation for vaccine authorization and assessment”, included four recommendations to ensure appropriate vaccine-specific expertise, tools, and resources are duly considered in the EU HTAR. This study contributes to this ongoing dialogue, by proposing strategies to strengthen vaccine assessment within the EU HTAR framework.

## 2. Materials and Methods

A targeted literature review and guideline analysis was conducted in December 2023 to identify the opportunities and challenges that NITAGs’ involvement would present to vaccine assessment and policymaking under the EU HTAR. The review focused on identifying relevant sources on the role of NITAGs, the EU HTAR, and vaccine-specific HTA methodologies. Searches were conducted via PubMed and Embase for peer-reviewed literature, while gray literature was identified through targeted searches of policy documents, official reports, implementing acts, and guidance documents from the EC, the HTA Coordination Group (HTA CG), the WHO, the ECDC, and national HTA bodies and NITAGs. Newly released guidance documents and implementing acts were incorporated throughout 2024 to ensure that the analysis remained up to date with evolving regulatory frameworks and vaccine-specific assessment requirements. To ensure a structured and comprehensive literature search, key search terms were developed using Boolean operators (AND, OR), incorporating both MeSH terms and free-text keywords. Searches included terms related to “Health Technology Assessment” AND “vaccines” OR “immunization” OR “vaccine evaluation”, as well as “National Immunization Technical Advisory Group” AND “policy recommendation” OR “scientific advice”. Additional terms captured aspects of “EU HTAR”, “Joint Clinical Assessment” and “horizon scanning”. Inclusion criteria focused on publications that addressed vaccine-specific HTA processes in the EU, NITAG roles, or relevant regulatory frameworks. Publications from 2016 to 2024 were included to ensure the most up-to-date coverage of the topic. Policy documents, reports, and empirical studies were of special interest.

The impact of implementing the EU HTAR on the diverse national HTA frameworks was investigated through a scenario-based case study of three MS, representative of the archetypal HTA systems of Europe (as identified in the 2021 paper by Laigle et al. [12]): Germany, where the NITAG is responsible for the initial clinical and sometimes economic vaccine assessment and recommendations for NIP; Italy, one of only four European countries where the HTAb is involved in the vaccine appraisal and a vaccine-specific evaluation framework is applied; and The Netherlands, where the HTAb is involved in vaccine assessment either before or in parallel with NITAG processes but where there is no vaccine-specific framework that is used by the HTAb.

The three-country case study compared the current national vaccine assessment processes with the timeline established by the EU HTAR to identify overlaps and discrepancies between established practice and the incoming joint assessment. The websites of key regulatory and advisory bodies related to HTA—such as the Italian Medicines Agency (AIFA), the Dutch Healthcare Institute (ZIN), the National Institute for Public Health and the Environment (RIVM of The Netherlands), the Paul-Ehrlich-Institut (PEI), Federal Joint Committee (G-BA), and Robert Koch Institute (RKI) in Germany—were reviewed to identify relevant documents and guidelines.

To address knowledge gaps regarding country-specific HTA processes and their timelines identified during the literature review, direct correspondence with these institutions was undertaken. Clarifications were sought regarding the frequency of horizon scanning and the role of manufacturers in initiating vaccine assessments. Responses from AIFA, ZIN, RIVM, PEI, and RKI provided critical insights and were integrated into the final analysis.

Building on the findings from the literature and case studies, together with the expertise of the Vaccines Europe Market Access working group, we presented our perspective on the current situation and developed strategic recommendations to support the objectives of the EU HTAR for vaccine assessment. These recommendations, considering both short-term and long-term outcomes, aim to address existing weaknesses and leverage strengths to ensure cohesive, consistent, and timely vaccine-specific HTA appraisals in the EU.

## 3. Results

### 3.1. The Role of NITAGs in Vaccine Market Access: Opportunities and Challenges

**Figure 1:**
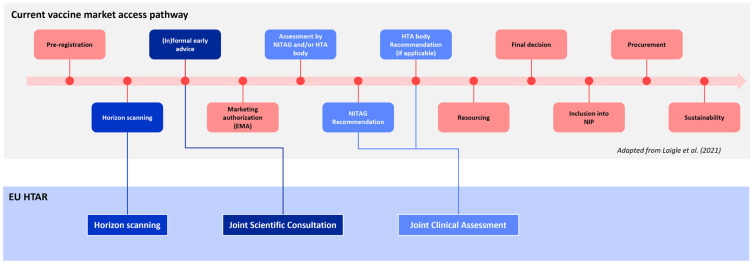
Timelines of the current vaccine market access pathway and the EU HTAR, with the different blue boxes highlighting the steps and the component of the EU HTAR that they overlap with [12].

Our literature review identified several key steps in the current vaccine market access pathway across EU countries. These steps are horizon scanning, early advice, initiation of assessment, NITAG recommendations for consideration of vaccines into the NIPs and funding, HTAb recommendation, final decision, NIP inclusion, and procurement [12]. As several steps of the EU HTAR overlap with the current vaccine market access pathways in EU countries, it is important to streamline the processes by leveraging the existing knowledge and expertise on vaccines, including that of NITAGs. Specifically, NITAGs, with their specialized knowledge and expertise, can play a critical role in building and enhancing these processes. Their involvement will ensure that none of the vaccine-specificities are overlooked and that all relevant stakeholders are effectively engaged in the joint HTA activities [12]. Figure 1 shows the different steps in the vaccine market access pathway, as described by Laigle et al. (2021) [12], with the different blue boxes highlighting the steps that overlap with the EU HTAR. The JCA process of the EU HTAR will run parallel to the EMA marketing authorization procedure, with certain deadlines being linked to key milestones of the EMA assessment. This process is intended to last about a year. However, the implications for the national HTA timelines are still unclear, as they do not always align fully with the expected EU HTAR timelines and are also not always fixed.

The implementation of the EU HTAR for vaccines from 2030 on currently overlooks some key vaccine-specific processes; in particular, the role of NITAGs. NITAGs play a pivotal role in providing evidence-based recommendations tailored to national immunization strategies, and the fact that they are currently not yet directly mentioned in the EU HTAR as well as its implementing acts or HTA CG guidance documents raises concerns regarding the comprehensiveness and consistency of vaccine assessments under the new framework [3,16,17,18,19,20]. A key consideration is the appointment of the assessor and co-assessor for JCAs. With only a limited number of HTAbs possessing expertise in vaccine assessment, the pool of potential assessors is restricted. Expanding the pool of assessors with vaccine expertise is essential to ensuring a balanced workload, as relying on a limited number of HTABs may lead to overburdening. Involving NITAGs in these roles could help distribute responsibilities more equitably, alleviating pressure on HTABs while strengthening the vaccine assessment process.

Without clear guidance on whether NITAGs will be involved, it remains uncertain who will fill this expertise gap. This ambiguity leaves the responsibility largely to individual MSs, potentially leading to significant discrepancies in how JCAs are conducted and utilized across EU countries. Such variability may undermine the harmonization goals of the HTAR and complicate efforts to create a unified approach to vaccine evaluation and market access.

Additionally, not involving NITAGs in the JSC could lead to disjointed approaches to vaccine development and assessment. As noted in the response from Vaccines Europe to the draft implementing act for the JSC for medicinal products, including NITAGs in JSC could foster greater alignment between the JSC, JCA and the national assessments. Not involving NITAGs in the JSC could lead to inefficiencies and potentially conflicting recommendations across MS [20]. This could also occur if NITAGs are not involved in the JCA.

Including NITAGs in the EU HTAR presents several opportunities for ensuring that accurate, evidence-based, independent vaccine expertise is included in the future joint clinical assessments. This inclusion could enhance efficiency at several key stages—horizon scanning, JSC, PICO scoping, and JCA—thereby streamlining processes, reducing duplication of work, and ensuring outcomes are transparent, applicable, and optimized to achieve the shortest possible timeline for population access to vaccines.

Alternatively, the challenges of not including NITAGs are not solely based on an expertise gap. NITAG roles, activities, and outcomes vary across the EU MS, and some, but not all, MSs also make use of HTAbs. This heterogeneity of national vaccine assessment processes involving NITAGs and/or HTAbs among EU MS suggests that the EU HTAR would not be equally and consistently applicable in each country. Additionally, the use of JCA reports at a national level may be limited where overlapping process steps lead to confusion or the doubling of work. Without including NITAGs in the EU HTAR and formal and structured communication between NITAGs and HTA CG/national HTAbs, the EU HTAR’s goal of accelerating time to patient access for vaccines could be hindered by unnecessary work duplication, further complexity and/or overlapping of processes, and extended timelines. These challenges present a real risk to the goal of the EU HTAR and to vaccine market access timelines. The identified opportunities and challenges are summarized in Table 1.

### 3.2. Case Studies: Vaccine Market Access in Germany, Italy, and The Netherlands

To assess the consequences of introducing the EU HTAR within the heterogeneous vaccine assessment frameworks of the EU MS, a case study of three countries representative of the archetypal HTA systems of Europe was performed. The case study of the three EU countries—Italy, The Netherlands, and Germany—highlighted key differences in their vaccine market access pathways. These include variations in the frequency of horizon scanning, the initiators of assessments, and the evidence bases informing NITAG recommendations. Across most EU MS, including the countries studied, NITAG recommendations are generally adopted by decision-makers, typically the Ministry of Health. Table 2 provides a comparative overview of the HTA approaches used in Italy, The Netherlands, and Germany, illustrating how each integrates national priorities and processes into vaccine evaluation and decision-making.

**Figure 2:**
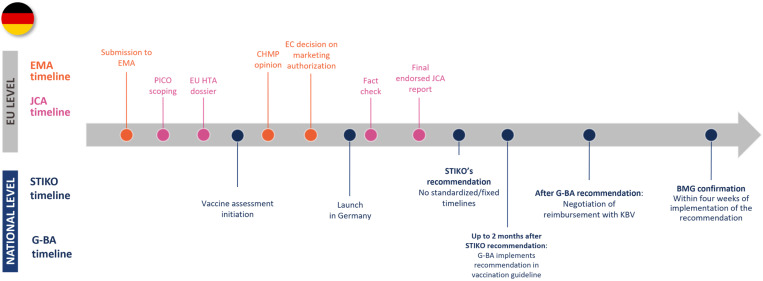
Timelines of EMA, JCA, and national bodies for Germany. Abbreviations: BMG—Bundesministerium für Gesundheit; CHMP—Committee for Medicinal Products for Human Use; EC—European Commission; EMA—European Medicines Agency; G-BA—Gemeinsamer Bundesausschuss; JCA—joint clinical assessment; KBV—Kassenärztliche Bundesvereinigung (Association of Statutory Health Insturance Physicians); STIKO—Ständige Impfkommission.

**Figure 3:**
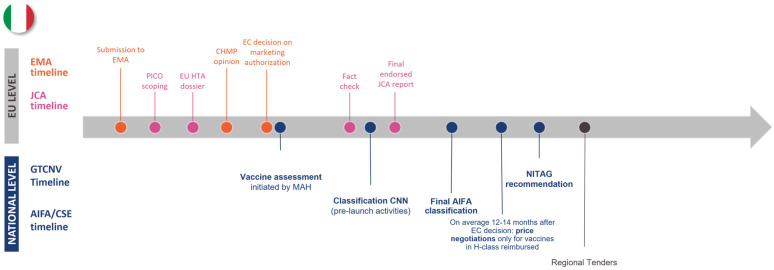
Timelines of EMA, JCA, and national bodies for Italy. Abbreviations: AIFA—Agenzia Italiana del Farmaco; CHMP—Committee for Medicinal Products for Human Use; CNN—class C non-negotiated; CSE—Commissione Scientifica ed Economica del Farmaco; EC—European Commission; EMA—European Medicines Agency; JCA—joint clinical assessment; MAH—market authorization holder; NITAG—National Immunization Technical Advisory Group.

**Figure 4:**
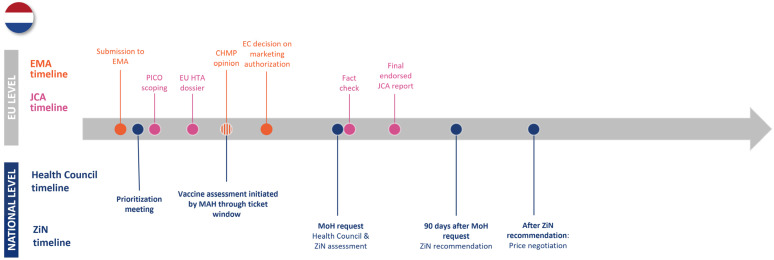
Timelines of EMA, JCA, and national bodies for The Netherlands. Abbreviations: CHMP—Committee for Medicinal Products for Human Use; EC—European Commission; EMA—European Medicines Agency; JCA—joint clinical assessment; MAH—market authorization holder; MoH—Ministry of Health; ZiN—Zorginstituut Nederland.

The case studies of Germany, Italy, and The Netherlands emphasized that national vaccine assessment timelines and processes often do not align neatly with the EU HTAR timelines and are subject to variation. Each of the three countries has NITAG involvement at the vaccine assessment point, but where this interaction occurs compared to the EMA and JCA timelines differs between countries. None of the countries started the NITAG recommendation at the same time as the start of the JCA report, as the NITAG recommendation takes place after the CHMP opinion, while the JCA report should be written before that (see Figure 2, Figure 3 and Figure 4). The German NITAG, the Ständige Impfkommission (STIKO—Standing Committee on Vaccination), generally provides recommendations for approved vaccines (i.e., assessment begins after the CHMP opinion), meaning there is a misalignment from the start between national and EU HTAR timelines [27,28,29]. In Italy, vaccine assessment is initiated by the market authorization holder once the EC’s decision is rendered; however, closer collaboration between the NITAG and HTAb during the evaluation process could help mitigate delays by ensuring streamlined communication through established national procedures [12,25]. In The Netherlands, the Zorginstituut Nederland (ZiN—National Health Care Institute) provides recommendations in parallel with the Gezondheidsraad (Health Council of The Netherlands) at the request of the Ministry of Health, though vaccine assessment for medical risk groups can begin upon request by the marketing authorization holder at the time of the CHMP opinion [30,31,32]. This means that, similarly to Germany, the national assessment will start after the first draft of the JCA report. For each of these countries, NITAG involvement would come after the JCA process, leading to duplication of work and lengthening timelines—or risking non-applicable or non-aligned JCA reports at a national level. Additionally, starting after the JCA report also poses some issues, as national authorities would need to wait for the JCA assessment before beginning their evaluations, potentially delaying the overall assessment process. Additionally, some authorities might postpone preparatory work until the JCA report is received, further extending timelines. Countries with pre-established schedules for vaccine assessments may also need to adjust their timelines to align with the JCA, creating potential disruptions. Only Italy, where the NITAG and HTAb collaborate to assess vaccines, would face little to no risk in not including NITAGs in the Regulation.

### 3.3. Recommendations and Potential Solutions for Involving NITAGs in the EU HTAR

With the EU HTAR having come into force on 12 January 2025, and as there is no clarity on considering vaccine specificities therein, an open discussion is needed to determine how vaccines will be evaluated under the EU HTAR. From this study, we developed the following recommendations (not prioritized):

- Structurally involve NITAGs in all activities of the HTA CG and clarify their position in national vaccine assessment and appraisal processes to ensure timely population access and the inclusion of relevant expertise.
- Address the unique assessment framework and expertise requirements of vaccines in the interim EU HTAR evaluation in 2028.
- Build a common understanding of the EU HTAR and its applicability to vaccines, provide training (e.g., rapid capacity building and more structured learning/development) for NITAG members as well as for vaccine assessors.
- Ensure that NITAGs provide input into the scoping process at a national level to optimize assessment scope applicability and add resources if needed.
- Consider NITAG members as assessors/co-assessors in the JCA process to ensure the appropriate expertise and quality.
- Incorporate the ECDC as a key stakeholder and partner to facilitate the implementation of the EU HTAR for vaccines. The Health Emergency Preparedness and Response Authority (HERA) and the EMA should also be involved [33].

## 4. Discussion

The EU HTAR builds on the established practice of evaluating vaccines alongside medicinal products, which is a long-standing approach in many countries. This provides a strong foundation but also presents an opportunity to further refine vaccine assessments and enhance access to innovative immunization strategies across the EU. Implementing a structured and transparent approach will help ensure that the unique characteristics of vaccines—such as indirect protection and public health benefits—are consistently recognized within the EU HTAR framework. By strengthening vaccine-specific expertise and improving alignment between EU and national processes, the framework can better support timely and effective vaccine access.

Article 4.1 from the EU HTAR states that when developing procedures, the HTA CG must take into account the particular characteristics of the health technology being assessed. This underscores the importance of ensuring that procedures are tailored to the unique aspects of each technology, including vaccines, throughout the evaluation process [13]. The case studies on Germany, Italy, and The Netherlands highlight areas where NITAGs and the HTA CG may have overlapping roles, particularly in areas like horizon scanning and JSCs. Strengthening communication on horizon scanning at the EU level with NITAGs could help minimize duplication of efforts and improve transparency, as NITAGs are not consistently involved in national horizon scanning initiatives. While not all NITAGs currently provide scientific advice, the EU HTAR presents an opportunity for them to integrate its outcomes into future assessments. Their involvement in JSCs could also enhance alignment between JSCs, joint clinical assessments (JCAs), and national processes, reducing redundancies. As only a limited number of EU MS use a decision-analytic framework, some NITAGs may not actively engage in JCA scoping. Without their involvement, JCA reports risk becoming misaligned with NITAG requirements for assessments and recommendations, e.g., by the inclusion of non-relevant PICOs in JCA reports. This could limit their usefulness for NITAGs and other decision-makers at national level [12,34]. The role of NITAGs in national assessments varies across Member States, meaning their exclusion from the EU HTAR could impact JCA alignment and applicability differently across countries. Furthermore, if NITAGs are not engaged from the outset, there is a greater risk of misinterpretation or underutilization of JCA reports at the national level. The case studies reinforce the need for robust communication and collaboration between NITAGs and the HTA CG subgroups. Establishing structured interaction channels at both national and EU levels will be key to ensuring a well-integrated and effective vaccine assessment process under the EU HTAR.

Building on the findings of this study, the expertise of the Vaccines Europe Market Access Working Group, and the perspectives outlined in this paper, several key recommendations emerge. These recommendations aim to optimize the integration of vaccine-specific clinical expertise within EU HTAR assessments. Additionally, they seek to ensure that the unique characteristics of vaccines—such as indirect protection and public health benefits—are consistently accounted for within a standardized vaccine framework endorsed by relevant parties and stakeholders. As Laigle and colleagues have demonstrated, there is considerable potential for improving alignment of vaccine evaluations across the EU [12]. The recommendations outlined in this paper represent potential strategies to enhance vaccine assessments under the EU HTAR; however, they have not yet been prioritized. The determination of their feasibility, relevance, and implementation timeline should be conducted in close cooperation with key stakeholders, including NITAGs, HTAbs, policymakers, and industry representatives. Active engagement with these stakeholders is essential to ensure that the recommendations are harmonized with existing frameworks, address real-world challenges, and accommodate the diverse needs of EU MS. Future discussions and cross-sector collaborations will be necessary to refine these recommendations, establish priorities, and develop a clear roadmap for their integration into the evolving EU HTA landscape.

The feasibility of implementing the recommendations put forward in this paper needs to be assessed, with the interim evaluation in 2028 providing a logical milestone. This timeline allows time for refining the EU HTAR’s provisions and developing vaccine-specific methodologies before the EU HTARs become applicable to vaccines in 2030. For these recommendations to be effectively implemented, it is crucial that all EU MS establish a NITAG if one is not already in place, and clearly defines its role and mandate. Without universal NITAG representation, there is a risk that countries without such advisory bodies may have limited influence in shaping the vaccine evaluation process under the EU HTAR. Establishing NITAGs across all Member States is therefore essential to promote equitable representation, consistency, and alignment in vaccine assessments across the EU.

These recommendations also incorporate findings from case studies in Germany, Italy, and The Netherlands, which represent three of the six country clusters identified by Laigle et al. (2021) [12]. While these countries provide valuable insights due to their well-established HTA frameworks, they may not fully capture the diversity of approaches across all EU Member States, particularly smaller countries or those with fewer resources. To enhance the understanding of the EU HTAR’s impact, future research should expand the scope of analysis to include a broader range of EU Member States. This could be achieved by utilizing both qualitative and quantitative methodologies to reflect the varied contexts, challenges, and opportunities across the EU. Additionally, the reliance on direct communications to fill information gaps introduces a limitation to the study’s reproducibility, as future research may not have access to the same institutional responses. Additionally, variations in institutional policies or personnel changes could affect the consistency of information obtained through such correspondence. And finally, we observed that while all three countries benefit from NITAG involvement, they each use distinct approaches. Greater alignment in these methods could enhance the overall efficiency and coherence of vaccine assessments within the EU. To improve alignment, we recommend fostering closer collaboration and sharing best practices between national NITAGs and HTA bodies. By standardizing certain processes, we can ensure that all countries consider vaccine-specific characteristics consistently. Furthermore, a more unified approach would reduce duplication of efforts, improve transparency, and strengthen the overall robustness of vaccine evaluations.

Engaging with relevant stakeholders will be vital while discussing and refining these recommendations, as well as exploring their roles in the evolving EU HTAR landscape. Capturing the full value and benefits of vaccines within the EU HTAR requires vaccine-specific methodologies, expertise, and resources. The next step will be to gather feedback from domain experts, including members of HTAbs and NITAGs, to further refine these recommendations. It is also imperative to establish alternative mechanisms for stakeholder engagement within the EU HTAR framework. Strengthening collaboration between EU and global vaccine advisory bodies—even without in-person meetings—could enhance consistency and robustness in vaccine evaluations across different regulatory landscapes.

Further research is needed to explore how NITAGs could play a role in other dimensions, such as the digital space. For instance, NITAGs could help ensure that the messages they communicate at both national and EU levels align with the JCA and horizon scanning processes. They could also translate complex JCA reports into more accessible language for non-expert audiences, thereby enhancing understanding among healthcare providers and the general public. Future studies should examine concrete mechanisms for NITAGs to engage with emerging digital tools and communication strategies, as this would not only strengthen their advisory role but also increase the overall impact and adoption of the EU HTAR.

## 5. Conclusions

Recognizing the complexity and specificity of vaccine assessment and formally including NITAGs in the EU HTAR will be key to achieving its objectives for vaccines. This includes streamlining national vaccine assessment processes, preventing duplicative efforts, supporting national authorities in making more informed and timely decisions, and fostering broad discussion among various stakeholders. Ultimately, this will help accelerate equitable population access to vaccination across EU Member States.

## Figures and Tables

**Table 1:** Opportunities and challenges arising from the involvement—or potential absence—of NITAGs in the EU HTAR.

Opportunities of Involving NITAGs	
Involving NITAGs in the new EU HTAR…	… ensures that expertise on vaccine-specific characteristics is incorporated during the joint clinical assessment.
	… secures that NITAGs can support the application EU HTAR by expanding the pool of available assessors.
	… components like joint horizon scanning across all EU MS would ensure a level playing field and allow each country’s health system to benefit from timely anticipation of new vaccines, enhance system-level preparedness and resilience, and address time-to-population access disparities while mitigating serious cross-border health threats.
	… components like JSCs enable NITAGs to anticipate innovative vaccines and advise developers on appropriate evidence-generation plans to increase the usability of JCAs at national level.
	… components like PICO scoping and JCAs ensure that the clinical HTA scope matches the needs of all EU MSs and minimizes the need for additional local data, reducing time to population access.
	… components like PICO scoping and JCAs ensure that the clinical HTA scope matches the needs of all EU MSs and minimizes the need for additional local data, reducing the time to population access.
**Challenges of not involving NITAGs**	
Not involving NITAGs in the new EU HTAR might…	… limit the use of JCA reports at the national level, risking duplicated efforts and hindering the HTAR’s goal of pooling resources and strengthening scientific quality of HTA across the EU.
	… complicate the EU HTAR instead of streamlining HTA methodologies across EU MS.
	… potentially lengthen the time required to access new vaccines, compromise population-wide coverage, and ultimately undermine efforts to safeguard public health.

Abbreviations: EU—European Union; HTA—health technology assessment; HTAR—regulation on health technology assessment; JCA—joint clinical assessment; NITAG—National Immunization Technical Advisory Group; PICO—Population Intervention Comparator Outcome.

**Table 2:** HTA approaches of the three case study countries for vaccine market access.

Vaccine Market Access Parameter	Germany	Italy	The Netherlands
Level of decision-making	National	National and regional	National
Body conducting vaccine assessment	NITAG (STIKO), assisted by the public health institute (RKI)	NITAG (GTCNV), HTAbs (AIFA/CSE)	NITAG (GR) in parallel with HTAb (ZiN)
Horizon scanning	Meeting with each manufacturer once per year by public health institute (RKI)	Meetings with manufacturers do take place, but without a fixed frequency (GTCNC)	Meeting with all manufacturers 4 times per year by public health institute (RIVM) [21]
Formal scientific advice	Regulatory authority (PEI) [22]	Not implemented (new scientific advice on track) [23]	ZiN, optionally in parallel with the regulatory body (CBG)
Manufacturer-initiated assessment	Not allowed (initiated by the STIKO and prioritized according to degree of public interest)	Allowed	Not allowed for GR assessment; allowed for ZiN assessment
NITAG recommendation basis	Clinical value, and benefit–risk assessment [24]	Clinical value, HTA presented by MAH, organization and financial impact [25]	Clinical value, appropriate use and efficiency [26]

Abbreviations: AIFA—Agenzia Italiana del Farmaco (Italian Medicines Agency); CBG—College ter Beordeeling van Geneesmiddelen (Medicines Evaluation Board); CSE—Commissione Scientifica ed Economica del Farmaco (Scientific and Economic Committee for Medicines); GR—Gezondheidsraad (Health Council of The Netherlands); GTCNV—Gruppo Tecnico Consultivo Nazionale Vaccinazioni (National Technical Advisory Group on Vaccinations); HTA—health technology assessment; HTAb—health technology assessment body; MAH—marketing authorization holder; NITAG—National Immunization Technical Advisory Group; PEI—Paul-Ehrlich Institute; RIVM—Rijksinstituut voor Volksgezondheid en Meilieu (National Institute for Public Health and the Environment); RKI—Robert Koch Institute; STIKO—Ständige Impfkommission (Standing Committee on Vaccination); ZiN—Zorginstituut Nederland (National Health Care Institute).
